# BET Bromodomain Blockade Mitigates Intimal Hyperplasia in Rat Carotid Arteries

**DOI:** 10.1016/j.ebiom.2015.09.045

**Published:** 2015-09-28

**Authors:** Bowen Wang, Mengxue Zhang, Toshio Takayama, Xudong Shi, Drew Alan Roenneburg, K. Craig Kent, Lian-Wang Guo

**Affiliations:** a Department of Surgery, Wisconsin Institute for Medical Research, Madison, WI 53705, USA; b University of Wisconsin Hospital and Clinics, University of Wisconsin School of Medicine and Public Health, Madison, WI 53705, USA

**Keywords:** IH, intimal hyperplasia, SMC, vascular smooth muscle cell, EC, vascular endothelial cells, BET, bromo- and extra-terminal domain family of epigenetic readers, BRD4, bromodomain-containing protein 4, a BET family member, JQ1(+), a BET-specific bromodomain inhibitor, JQ1(−), inactive enantiomer, PDGF, platelet-derived growth factor, Intimal hyperplasia, Smooth muscle cell, Epigenetic reader, BET, BRD4

## Abstract

**Background:**

Intimal hyperplasia is a common cause of many vasculopathies. There has been a recent surge of interest in the bromo and extra-terminal (BET) epigenetic “readers” including BRD4 since the serendipitous discovery of JQ1(+), an inhibitor specific to the seemingly undruggable BET bromodomains. The role of the BET family in the development of intimal hyperplasia is not known.

**Methods:**

We investigated the effect of BET inhibition on intimal hyperplasia using a rat balloon angioplasty model.

**Results:**

While BRD4 was dramatically up-regulated in the rat and human hyperplastic neointima, blocking BET bromodomains with JQ1(+) diminished neointima in rats. Knocking down BRD4 with siRNA, or treatment with JQ1(+) but not the inactive enantiomer JQ1(−), abrogated platelet-derived growth factor (PDGF-BB)-stimulated proliferation and migration of primary rat aortic smooth muscle cells. This inhibitory effect of JQ1(+) was reproducible in primary human aortic smooth muscle cells. In human aortic endothelial cells, JQ1(+) prevented cytokine-induced apoptosis and impairment of cell migration. Furthermore, either BRD4 siRNA or JQ1(+) but not JQ1(−), substantially down-regulated PDGF receptor-α which, in JQ1(+)-treated arteries versus vehicle control, was also reduced.

**Conclusions:**

Blocking BET bromodomains mitigates neointima formation, suggesting an epigenetic approach for effective prevention of intimal hyperplasia and associated vascular diseases.

## Highlights

• Blocking BET epigenetic readers with JQ1(+) mitigates neointimal proliferation in balloon-injured rat carotid arteries.
• JQ1(+) or BRD4 knockdown inhibits vascular smooth muscle cell proliferation, migration, and PDGF receptor expression.
• JQ1(+) prevents inflammatory dysfunction of vascular endothelial cells.

The transition of vascular smooth muscle cells to a migratory proliferative state produces a new thick layer of tissue on the inner vessel wall obstructing blood flow. Epigenetic control of this transition is poorly understood. We find that inhibiting a family of epigenetic regulators called “readers” halts this disease-prone process. Our study may open fresh opportunities for epigenetic interventions to prevent smooth muscle cell instability and associated occlusive vascular diseases that pose a great threat to public health.

## 1 Introduction

Intimal hyperplasia (IH) produces vascular lumen re-narrowing or restenosis, leading to failure of angioplasty or bypass commonly performed to treat cardiovascular disease. Without preventive intervention the incidence of restenosis can reach ~ 30–50% within 6 months after surgery (Jukema et al., 2012). While the etiology of IH is multifactorial, at its center is the transition of vascular smooth muscle cells (SMCs) from quiescence to a proliferative and migratory cell state (Alexander and Owens, 2012). It has become recognized that this SMC phenotypic transition or switching is prompted by abnormalities in chromatin remodeling (Alexander and Owens, 2012). Aside from epigenetic writers (histone acetyl transferases) and erasers (histone deacetylases), there has emerged a new family of epigenetic readers, the bromo and extra-terminal domain (BET) proteins (Gillette and Hill, 2015).

The BET family comprises BRDT, BRD2, BRD3, and BRD4 (Shi and Vakoc, 2014). BRDT is testis-specific and hence irrelevant in the vascular system. Each BET protein contains tandem bromodomains that “read” (or bind) acetylated histones. In contrast to BRD2 and BRD3, BRD4 is unique in that it has a C-terminal domain to recruit the positive transcription elongation factor (p-TEFb) to paused RNA polymerase II activating transcriptional elongation (so-called pause release) (Shi and Vakoc, 2014, Kanno et al., 2014). Thus BRD4 “translates” chromatin remodeling to gene transcription.

BET epigenetic readers were traditionally deemed undruggable. However, a recent serendipitous discovery of a BET-specific bromodomain blocker (JQ1(+)) (Filippakopoulos et al., 2010), and subsequently its derivatives, have dramatically changed this view(Wang and Filippakopoulos, 2015). Intriguingly, blockade of BRD4 — a general transcription co-activator, does not suppress gene transcription globally, but rather, selectively inhibits the expression of a subset of overactive genes that are often associated with disease states (Wang and Filippakopoulos, 2015, Anand et al., 2013). The key of this selectivity may lie in clusters of transcription enhancers termed super-enhancers (Loven et al., 2013). Upon pathogenic stimulation, super-enhancers re-assemble at a defined set of genes and recruit BRD4 for transcriptional activation of these genes driving cell state transition (Amaral and Bannister, 2014, Brown et al., 2014). This notion has gained strong support from chemical genetic studies using JQ1(+) to inhibit cancer cell proliferation (Asangani et al., 2014, Loven et al., 2013), cardiomyocyte hypertrophy (Anand et al., 2013), and endothelial cell inflammation (Brown et al., 2014).

The primary cause of IH is the phenotypic transition of SMCs from a quiescent to a proliferative/migratory cell state, which involves active transcription of a subset of genes, such as growth factors and cytokines and/or their receptors (Alexander and Owens, 2012). We thus hypothesized that the BET epigenetic readers may play a critical role in this cell state transition (Wang et al., 2015), in particular, activation of the platelet-derived growth factor (PDGF) pathway — the most potent stimulant for vascular SMC proliferation and migration (Heldin and Westermark, 1999). Blocking BET bromodomains with JQ1(+) has exhibited high preclinical efficacy in a growing number of malignancies (Zuber et al., 2011, Delmore et al., 2011, Brown et al., 2014, Anand et al., 2013). However, the function of the BET family in restenotic vascular disease is not known. This study aimed to assess whether the BET family plays an important role in the development of IH.

## 2 Methods

### 2.1 Rat Carotid Artery Balloon Angioplasty Model and Peri-adventitial Administration of JQ1(+)

All animal studies conform to the *Guide for the Care and Use of Laboratory Animals* (National Institutes of Health publication No. 85–23, 1996 revision) and protocols approved by the Institutional Animal Care and Use Committee at the University of Wisconsin. Institutional review board (IRB) approval has been obtained for use of human samples.

### 2.2 Rat Carotid Artery Balloon Angioplasty and Peri-adventitial JQ1(+) Administration

Carotid artery balloon angioplasty was performed in male Sprague–Dawley rats (Charles River; 300–330 g) as previously described (Guo et al., 2014). Briefly, rats were anesthetized with isoflurane (5% for inducing and 2.5% for maintaining anesthesia). A longitudinal incision was made in the neck and carotid arteries were exposed. A 2-F balloon catheter (Edwards Lifesciences, Irvine, CA) was inserted through an arteriotomy on the left external carotid artery. To produce arterial injury, the balloon was inflated at a pressure of 2 atm and withdrawn to the carotid bifurcation and this action was repeated three times. The external carotid artery was then permanently ligated, and blood flow was resumed.

Balloon angioplasty was performed for two sets of experiments. (1) *Time course*. The left carotid arteries were collected at days 3, 7, and 14 post-angioplasty for immunostaining of BRD4; the uninjured right carotid arteries served as control. (2) *JQ1*(+) *treatment*. Immediately following angioplasty, JQ1(+)(CAS 1268524-70-4, Cayman Chemical, MI) (For compound structure, see publication) (Filippakopoulos et al., 2010) or DMSO (vehicle control) dissolved in F-127 Pluronic gel was applied to the outside of the balloon-injured segment of the carotid artery (Guo et al., 2014). For administration of JQ1(+), we used two doses (100 μg or 500 μg per rat) (Wang et al., 2015) which are believed to be well below toxic levels because even a dose of 50 mg/kg body weight did not show toxicity in previous studies (Asangani et al., 2014). JQ1(+) from a DMSO stock was dissolved in 25% F-127 Pluronic gel (Sigma-Aldrich) to a final volume of 300 μl (JQ1 concentration in the gel: 0.72 mM or 3.6 mM), which was then applied to the outside of the injured segment of the carotid artery. In the control group, equal volume of DMSO vehicle mixed with Pluronic gel was applied. This perivascular approach has been proven effective in the delivery of small-molecule drugs or even nanoparticles into the injured arterial wall (Shi et al., 2014). Two weeks after balloon angioplasty, common carotid arteries were collected from anesthetized animals (under 2.5% isoflurane) following perfusion fixation at a physiological pressure of 100 mm Hg (Guo et al., 2014). The animals were then euthanized in a CO_2_ chamber.

### 2.3 Morphometric Analysis of Intimal Hyperplasia (IH)

Paraffin sections (5 μm thick) were excised from carotid arteries at equally spaced intervals and then Van Gieson stained for morphometric analysis, as described in our previous reports (Guo et al., 2014). Planimetric parameters as follows were measured on the sections and calculated using Image J: area inside external elastic lamina (EEL area), area inside internal elastic lamina (IEL area), lumen area, intima area (= IEL area – lumen area), and media area (= EEL area – IEL area). Intimal hyperplasia was quantified as a ratio of intima area versus media area. Measurements were performed by a student blinded to the experimental conditions using 3–6 sections from each of 4 rats treated with either vehicle (DMSO) or JQ1(+). The data from all sections were pooled to generate the mean for each animal. The means from all the animals in each treatment group were then averaged, and the standard error of the mean (SEM) was calculated.

### 2.4 Immunohistochemistry for Assessment of BRD4, PDGFRα, and PCNA Expression in Rat and Human Arteries and Veins

**Fig. 1:**
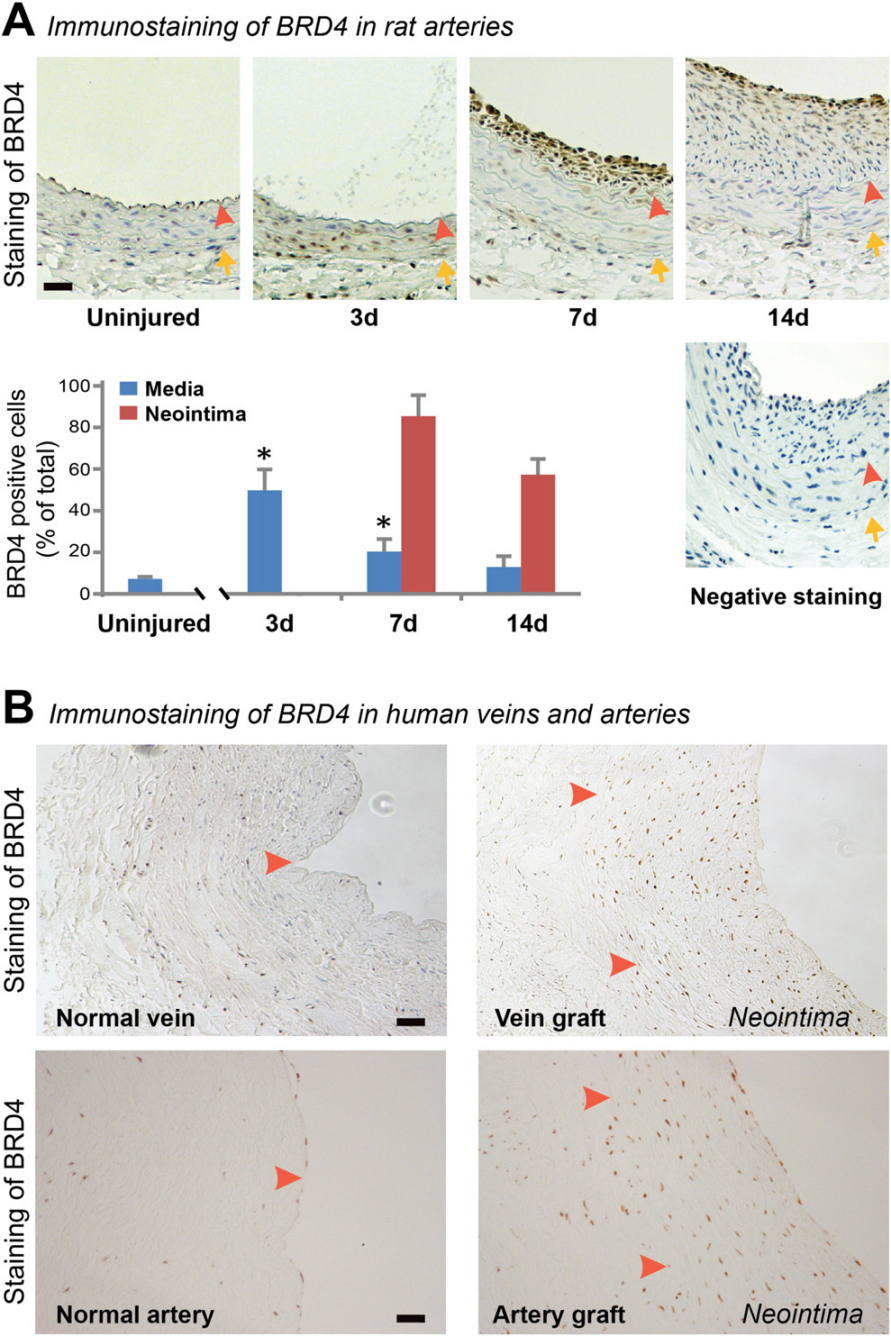
Up-regulation of BRD4 in the neointima of injured rat carotid arteries and in human vein and artery grafts. A. Immunostaining of rat carotid artery sections showing increase of BRD4 positive cells at days 3, 7, and 14 after balloon angioplasty versus uninjured arteries. “Negative staining” refers to background control without using a primary antibody. Quantification: mean ± SEM; n = 3 animals at each time point; *P < 0.05 compared to uninjured control. Arrowhead: internal elastic lamina (IEL); arrow: external elastic lamina (EEL). Neointima is defined between lumen and IEL. Scale bar: 50 μm. B. Immunohistochemistry showing increase of BRD4 in the neointima of human saphenous vein and internal mammary artery grafts versus normal vessels. Arrowhead: IEL. Scale bar: 50 μm.

Immunostaining was performed on rat carotid artery sections following our published methods (Guo et al., 2014). Briefly, the sections were first incubated with each of the primary antibodies for 1 h with a dilution ratio as follows: rabbit anti-BRD4 (Abcam, Cambridge, MA), 1:150; rabbit anti-PDGFRα (Bolster), 1:200; rabbit anti-PCNA (Santa Cruz), 1:200. The sections were then incubated with the ImmPRESS HRP-conjugated goat-anti-rabbit secondary antibody (Vector Laboratories, 1:200), followed by visualization with 3, 3-diaminobenzidine (DAB). The slides were counterstained with hematoxylin. The number of positively stained cells was counted on 8-bit binary images converted (by Image J) from the pictures of immunostained sections and normalized by the high power microscopic field (HPF). Cell counting was performed by a student blinded to experimental conditions. In each experimental group (DMSO control or JQ1(+) treatment), at least 8 sections from each of 4 animals were used. The data from all sections were pooled to generate the mean for each animal. The means from all the animals in each experimental group were then averaged, and the standard error of the mean (SEM) was calculated. The same immunohistochemistry conditions were applied to the human artery and vein samples. Hematoxylin counter-staining is not presented in Fig. 1 because we have found that intense counter-staining (blue) tends to overwrite the BRD4 immunostaining (brown) which is also localized in the nucleus.

### 2.5 Primary Vascular Smooth Muscle Cell and Endothelial Cell Cultures

Rat aortic vascular SMCs were isolated from the thoracoabdominal aorta of male Sprague–Dawley rats based on a protocol using an enzymatic dissociation method and cultured in Dulbecco modified Eagle low glucose medium (Gibco, Carlsbad, CA) containing 10% fetal bovine serum (FBS) (Guo et al., 2014). Human primary aortic smooth muscle cells and human primary aortic endothelial cells were purchased from Lonza and cultured in SmGM-2 complete medium containing 5% FBS and EGM-2 complete medium containing 2% FBS, respectively (Goel et al., 2014). Cells between passage 5 and 7 were used for all experiments and maintained at 37 °C with 5% CO_2 (__Guo et al., 2014__)_. For cell culture expansion, 0.25% Trypsin was used for detachment of rat and human SMCs, while Accutase (Lifetechnologies, Carlsbad, CA) was used for human ECs.

### 2.6 Cell Proliferation/Viability Assay for Rat and Human Aortic SMCs

To study the effect of JQ1(+) on the proliferation of rat as well as human aortic SMCs, we used both a Cell Proliferation BrdU ELISA (colorimetric) Kit (Roche Applied Science, Indianapolis, IN) and a CellTiter-Glo Luminescent Cell Viability kit (Promega, Madison, WI) following manufacturer instructions. SMCs were seeded in 96-well plates at a density of 4000 cells (BrdU assay) or 2000 cells (CellTiter-Glo assay) per well with a final volume of 200 μl, and starved for 24 h in DMEM (for rat cells) or SmBM-2 basal medium (for human cells) containing 0.5% FBS. Cells were then pretreated with a series of concentrations of JQ(+)1, JQ(−)1, or an equal volume of vehicle control (DMSO) for 1 h in fresh starvation medium prior to PDGF-BB (human recombinant, R&D Systems Inc., MN) mitogenic stimulation (final 10 ng/ml). After PDGF-BB treatment for 22 h, cells were labeled with BrdU by a 2-h incubation at 37 °C, and then fixed with a FixDenat solution for 30 min, followed by a 90-min incubation at room temperature with an anti-BrdU-POD antibody (1:100 dilution). After washing with PBS for 3 times, substrate was added. Plates were incubated at room temperature for 30 min, and then colorimetric signals were measured on a FlexStation 3 Benchtop Multi-Mode Microplate Reader (Molecular Devices, Sunnyvale, CA) at 370 nm with a reference wavelength of 492 nm.

Cell viability assay was performed as described in our previous report (Guo et al., 2014). Briefly, 96 h after PDGF-BB treatment, plates were decanted and re-filled with 50 μl CellTiter-Glo reagent/50 μl PBS per well. Plates were incubated at room temperature for 10 min and then read in a FlexStation 3 Benchtop Multi-Mode Microplate Reader (Molecular Devices, Sunnyvale, CA) using a 250 ms integration.

### 2.7 Scratch and Transwell Migration Assays

Scratch (wound healing) assay was performed as described in our previous report (Guo et al., 2014). Briefly, SMCs were cultured to a 90% confluency in 6-well plates and then starved for 24 h in DMEM medium containing 0.5% FBS, which was supplemented with 1 μM JQ1(+),1 μM JQ1(−), or equal volume of DMSO. A sterile pipette tip was used to generate an ~ 1 mm cell-free gap. Dislodged cells were washed away with PBS. Plates were then refilled with medium containing 10 ng/ml PDGF-BB and incubated for 24 h. At the end of the treatment, for illumination of the cells, Calcein AM was added at a final concentration of 2 μM and incubated for 30 min. After 3 times of wash with PBS, images were taken, and cell migration was quantified by Image J based on the change in the width of the cell-free gap before and after PDGF-BB stimulation. For Transwell assay, SMCs were starved for 24 h in DMEM medium containing 0.5% FBS, and then seeded at a density of 20,000/well in the upper chamber of Transwell Permeable Supports (or Inserts) (8 μm pore size, Corning, NY) placed in 24-well plates. Cells were pre-incubated with 1 μM JQ1(+), JQ1(−), or vehicle (DMSO) for 24 h prior to the stimulation of chemotaxis with 10 ng/ml PDGF-BB, which was added to the lower chamber at a final concentration of 10 ng/ml. Inserts were harvested 24 h post stimulation and fixed in 70% ethanol at − 20 °C for 30 min. Pre-wetted cotton swaps were used to gently scrape remaining cells in the upper chamber of inserts, followed by staining the cells on the lower surface of the insert in hematoxylin solution for 30 min at room temperature. The upper chamber of the inserts was swapped again and rinsed in PBS twice. After air-drying the inserts for 30 min, the polyester membranes were harvested using a scalpel and mounted on glass slides using 90% glycerol. Images were then taken for quantification of the cells that migrated from the upper chamber across the membrane to the lower surface.

### 2.8 Caspase-Glo 3/7 Assay for Endothelial Cell Apoptosis

Caspase-Glo 3/7 assay (Promega, Madison, WI) was used to determine the relative activity of caspase-3/7 according to manufacturer's instructions. Briefly, human aortic endothelial cells were seeded in 96-well plates at a density of 8000 cells/well and starved in 1% FBS medium for 24 h. Before stimulation with human recombinant TNFα or IL-1β (R&D Systems)(final concentration 20 ng/ml), cells were pretreated with 100 nM JQ1(+) or vehicle control for 24 h. Four hours after cytokine treatment, plates were decanted and re-filled with 50 μl Caspase-Glo 3/7 reagent and 50 μl PBS per well. Plates were incubated at room temperature for 1 h and then read in a FlexStation 3 Benchtop Multi-Mode Microplate Reader (Molecular Devices, Sunnyvale, CA).

### 2.9 siRNA Knockdown

Lentiviruses for expression of scrambled or rat BRD-specific siRNAs were packaged using a three-plasmid expression system (piLenti-siRNA-GFP, psPAX2 and pMD2.G). The piLenti-siRNA-GFP vectors for expression of a scrambled siRNA or an siRNA specific for rat BRD2, BRD3, or BRD4 were purchased from Applied Biological Materials Inc. (Canada). The target sequence of the most efficient BRD4 siRNA is GCGAATCTAGCTCCTCTGACAGTGAAGAC. For each of BRD2 and BRD4 two siRNAs were used. BRD2 siRNA#134: CCACAATGGC TTCTGTACCAGCTTTACAA; #1141: CCACAATGGCTTCTGTACCAGCTTTACAA. BRD3 siRNA #295: AGTGAGTGTAT GCAGGACTTCAACACCAT; #355: ACAGATGACATAGT GCTAATGGCCCAGGC. The psPAX2 and pMD2.G plasmids were kindly provided by Dr. Ming-Liang Chu (Guizhou Renmin Hospital, China). The 3 plasmids were co-transfected into HEK293T cells in DMEM medium containing 10% FBS using a JetPrime Polyplus-transfection reagent (Polyplus-transfection Inc., New York, NY) following the manufacturer's protocol. After transfection for 24 h, the medium containing transfection reagents was replaced with fresh DMEM medium containing 10% FBS. The culture medium was collected after 24 h incubation and the collections containing packaged lentivirus were passed through a 0.45 μm filter (EMD Millipore, MA), and then concentrated and titrated using Lenti-X™ Concentrator and Lenti-X™ qRT-PCR Titration Kit (Clontech Laboratories, Inc., Mountain View, CA), respectively. The lentivirus preparation was then applied to the SMC culture together with 8 μg/ml polybrene. Infected cells were recovered in fresh DMEM medium containing 10% FBS for 24 h and then used in experiments of proliferation or Western blotting.

### 2.10 Real-time Quantitative PCR (qRT-PCR)

mRNA was isolated from collected cells using TRIzol (Qiagen, Valencia, CA) following the manufacturer's instructions. Purified mRNA (1 μg) was used for the first-strand cDNA synthesis using iScript cDNA synthesis kit (Bio-Rad) and quantitative RT-PCR was performed using the 7500 Fast Real-Time PCR System (Applied Biosystems, Carlsbad, CA). Each cDNA template was amplified in triplicates using SYBR Green PCR Master Mix (Applied Biosystems, Carlsbad, CA) with the following primers: Rat BRD4, Forward CTGCCAGTAATGGGGGATGG, Reverse ATTGGTGCTGGCTGCATTTG. Rat BRD3, Forward TCCAGGATCAGCTGTTGAATGT, Reverse GCTGTGGTTGTGCTATTGGC. Rat BRD2, Forward GGGTCTGCCGGATTATCACA, Reverse GCCCCCTTCTTATGGCTGTT. Rat PDGFRα, forward GCC TCC ATT CTG GAG CTT GT, reverse AGC TCT CTG TTC CCA ATG CC. Rat PDGFRβ, forward ATG AGT CAT CTC GGA CCC CA, reverse TCA GGG GCA GAT GGG ACA TA. Rat GAPDH, forward GAC ATG CCG CCT GGA GAA AC, reverse AGC CCA GGA TGC CCT TTA GT.

### 2.11 Western blotting for Evaluation of Protein Levels

SMCs were lysed in RIPA buffer containing protease inhibitors (50 mM Tris, 150 mM NaCl, 1% Nonidet P-40, 0.1% sodium dodecyl sulfate, and 10 μg/ml aprotinin). Protein concentrations of cell lysates were determined using a Bio-Rad DC™ Protein Assay kit. Approximately 15–30 μg of proteins from each sample were separated on 4–20% Mini-PROTEAN TGX precast gels (Bio-Rad) and transferred to a PVDF membrane. Proteins of interest were detected by immunoblotting using the following primary antibodies and dilution ratios: Rabbit anti-BRD4 (1:1000) from Abcam (ab128874), rabbit anti-PDGFRα (1:1000) from Cell Signaling technology (3164 s), rabbit anti-PDGFRβ (1:200) from Santa Cruz (sc-432) and mouse anti-β-actin from Sigma-Aldrich. After incubation of the blots with HRP-conjugated secondary antibodies (1:3000 for goat anti-rabbit or 1:10,000 for goat anti-mouse, Bio-Rad), specific protein bands on the blots were visualized by applying enhanced chemiluminescence reagents (Pierce) and then recorded with a LAS-4000 Mini imager (GE, Piscataway NJ). Band intensity was quantified using ImageJ.

### 2.12 Statistical Analysis

Data are presented as mean ± standard error of the mean (SEM). Statistical analysis was conducted using two-tailed unpaired Student's t-test. Data are considered statistically significant when a P value is < 0.05.

## 3 Results

### 3.1 Epigenetic Reader BRD4 is Dramatically Up-regulated in the Neointima of Injured Rat Carotid Arteries as well as Human Vein and Artery Grafts

BRD4 is the best characterized BET member and has been shown to play a pivotal role in several disease states (Anand et al., 2013, Wang and Filippakopoulos, 2015). It is a unique BET member in that it is associated with super-enhancers and its C-terminal domain facilitates the recruitment of the positive transcription elongation factor p-TEFb to activate RNA polymerase II (Anand et al., 2013, Kanno et al., 2014). We thus set out to investigate the role of BET family in intimal hyperplasia (IH) by assessing BRD4 expression in the rat carotid artery in a balloon angioplasty model (Guo et al., 2014). While the ratio of BRD4-positive versus total cells in the media of uninjured arteries remained at a basal level of ~ 10%, this ratio increased to 50% at day 3 after balloon injury (Fig. 1A). Interestingly, while the expression of BRD4 in the media declined after day 3, concomitantly BRD4-positive cells dramatically increased in the neointima with a ratio of 85% versus total cells at day 7, and remained at 55% at day 14. A pronounced increase of BRD4 was also observed in the neointima of human saphenous vein and internal mammary artery grafts versus normal vessels lacking neointima (Fig. 1B and supplemental Figure S1), suggesting clinical relevance of our findings. These results demonstrate that the epigenetic reader BRD4 is dramatically up-regulated following the development of neointima in rat as well as human blood vessels, implicating its involvement in the process of IH.

### 3.2 Blocking BET Bromodomains with JQ1(+) Effectively Mitigates IH in Balloon-injured Rat Carotid Arteries

**Fig. 2:**
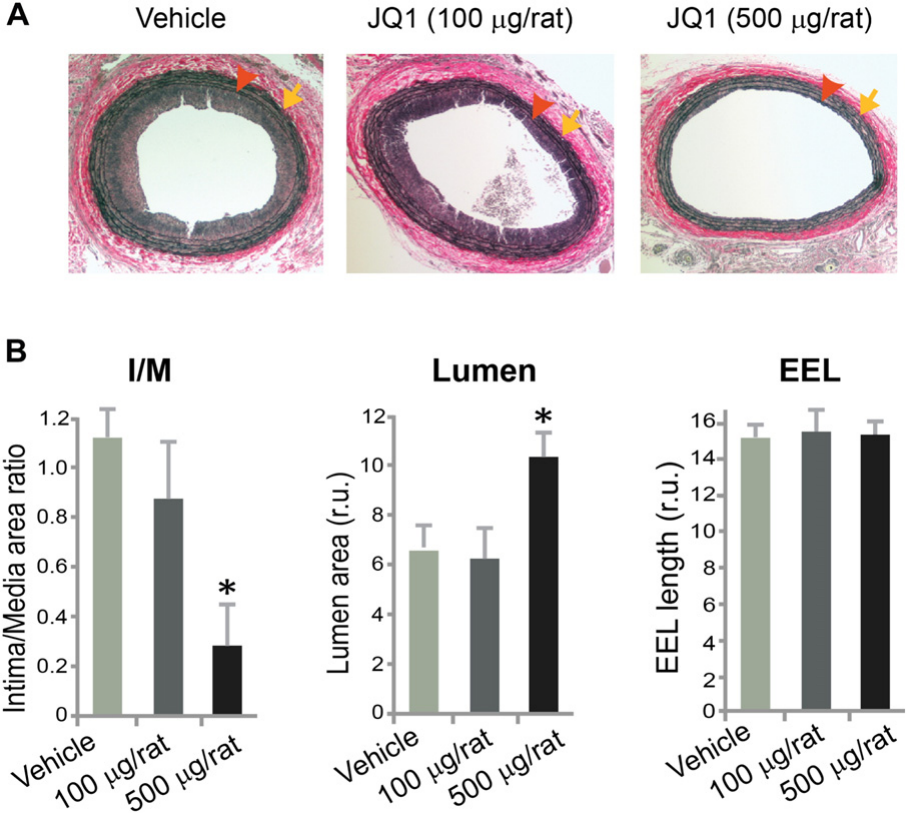
Inhibition of intimal hyperplasia by treatment with JQ1(+) in balloon-injured rat carotid arteries. A. Van Gieson-stained sections showing reduced neointima in JQ1(+)-treated rat carotid arteries compared to vehicle control 14 days after injury. Arrowhead: IEL; arrow: EEL. B. Quantification of IH (intima/media area ratio), lumen area, and EEL length is presented as mean ± SEM; n = 4 animals; *P < 0.05 versus vehicle control.

We then investigated the function of the BET family in the development of IH using the first-in-class BET-specific inhibitor, JQ1(+), which was recently discovered (Filippakopoulos et al., 2010). An acetyl lysine-binding pocket in the bromodomain is unique to the BET proteins. JQ1(+) occludes this pocket by mimicking the acetyl moiety and is thus highly specific for the BET family (Kanno et al., 2014). In our study (Fig. 2), treatment by peri-adventitial administration of JQ1(+) (500 μg per rat) reduced IH (I/M ratio) by 75% and enlarged the lumen by 60% versus vehicle control at day 14 post injury. A lower dose (100 μg per rat) produced a numerical decrease of IH which was not statistically significant. The vessel size measured by EEL (external elastic lamina) length was unaltered with either dose. These results demonstrate that blocking BET epigenetic reader activity can effectively mitigate IH, indicating an important role of this family in the formation of hyperplastic neointima.

### 3.3 BET Inhibition Mitigates SMC Proliferation and Migration In Vitro as well as In Vivo

**Fig. 3:**
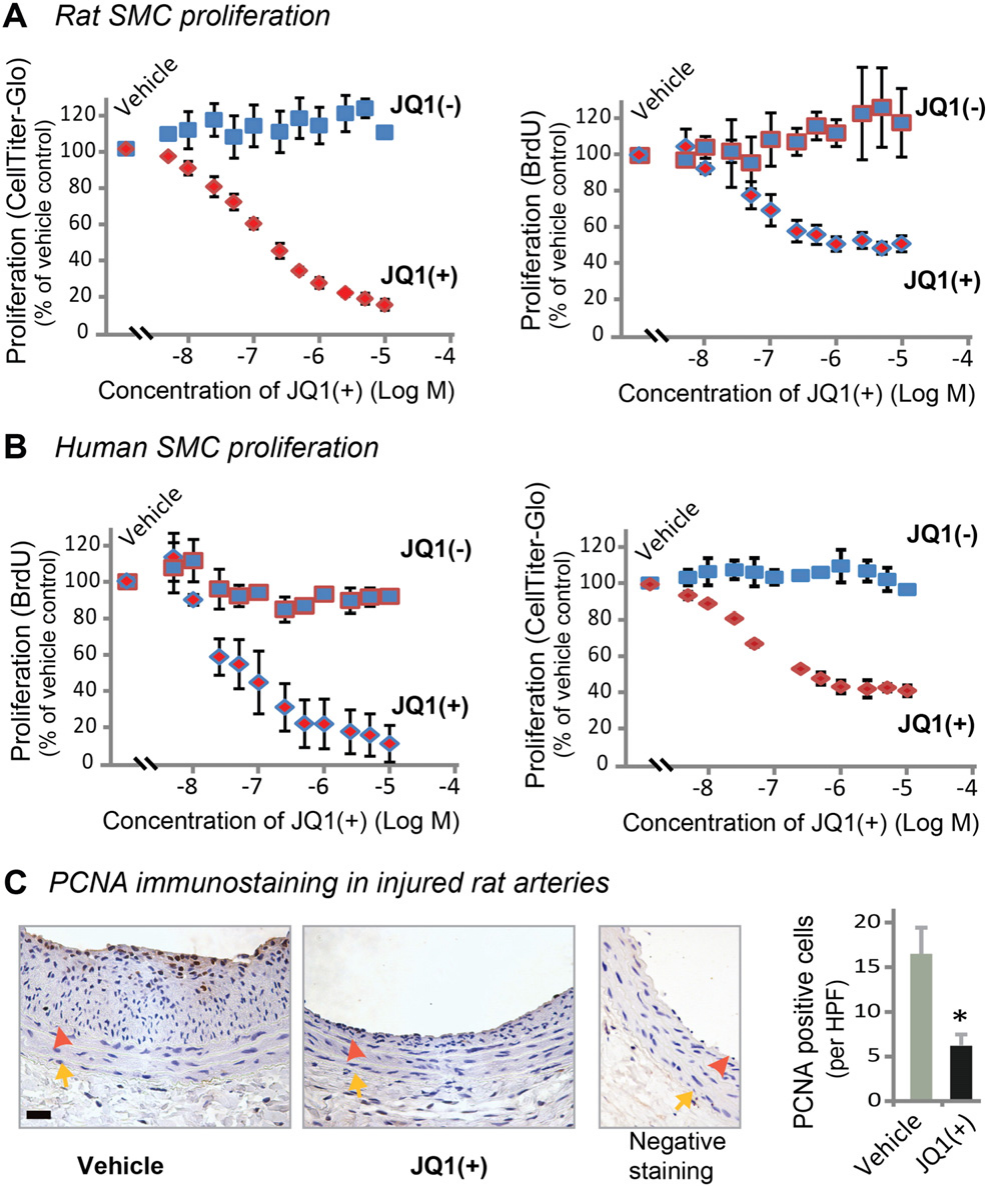
Inhibition of rat and human aortic smooth muscle cell proliferation by treatment with JQ1(+). A and B. Dose responses to JQ1 enantiomers showing inhibition of proliferation of primary rat (A) and human (B) aortic SMCs, by JQ1(+) but not JQ1(−). Proliferation was measured by CellTiter-Glo assay or BrdU assay 24 h after PDGF-BB stimulation. Quantification: mean ± SEM; n = 3 independent experiments. C. Immunostaining of PCNA showing reduced proliferating cells in JQ1(+)-treated (500 μg per rat) carotid arteries versus vehicle control 14 days after injury. Arrowhead: IEL; arrow: EEL. “Negative staining” refers to the background control without using a primary antibody. Quantification: mean ± SEM; n = 4 animals; *P < 0.05 versus vehicle control. HPF: high power field. Scale bar: 50 μm.

**Fig. 4:**
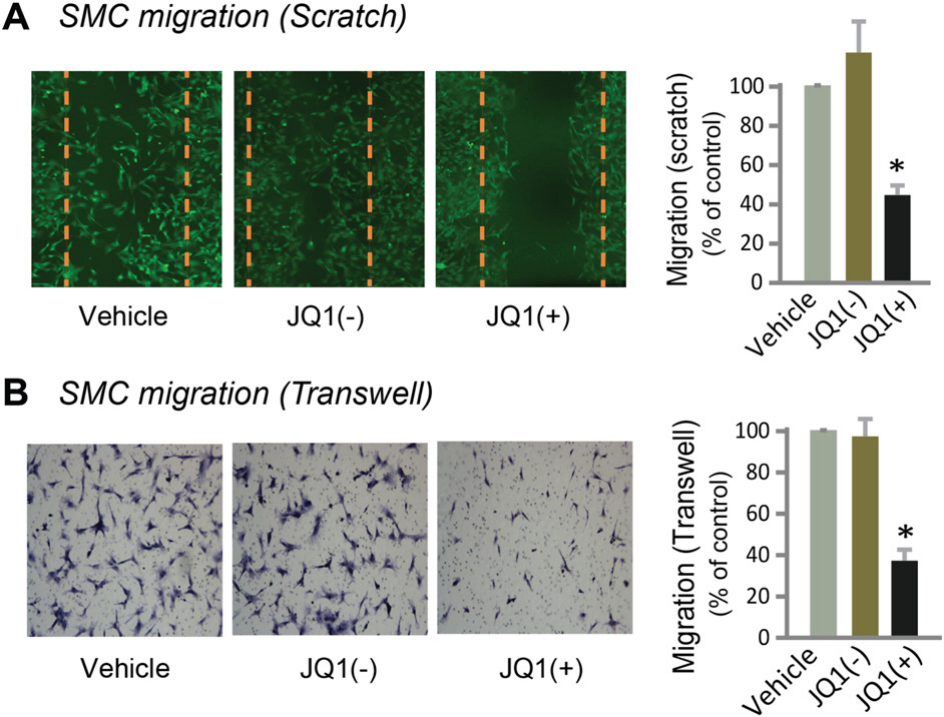
Inhibition of rat smooth muscle cell migration by treatment with JQ1(+). Scratch assay (A) and Transwell assay (B) showing inhibition of rat SMC migration by 1 μM JQ1(+) but not JQ1(−) at 24 h after PDGF-BB stimulation. Dashed line marks the cell-free gap at 0 h after scratch. Quantification: mean ± SEM; n = 3; *P < 0.05 versus vehicle control.

**Fig. 5:**
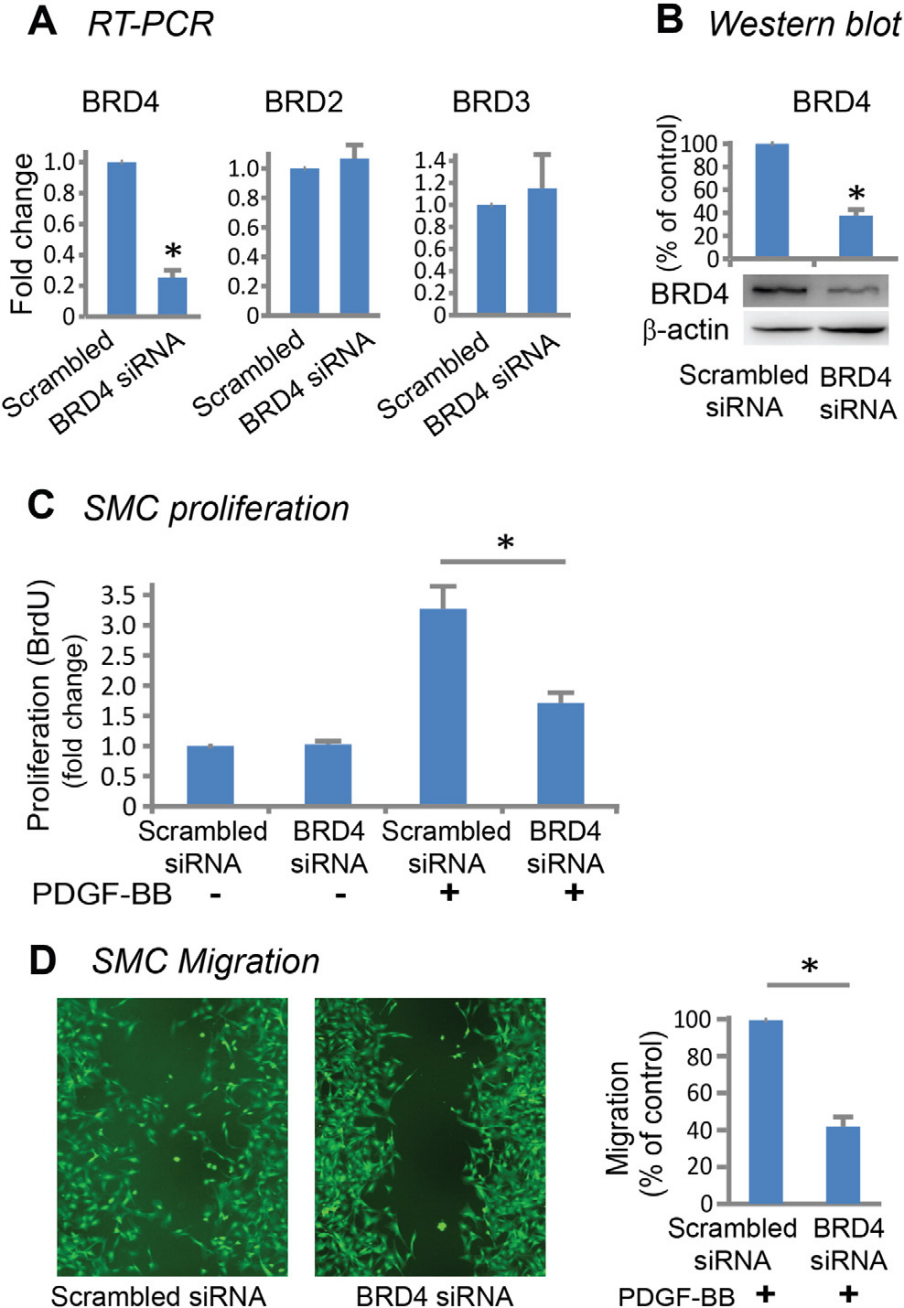
Inhibition of rat smooth muscle cell proliferation and migration by siRNA knockdown of BRD4. A and B. Specific siRNA knockdown of BRD4 indicated by RT-PCR (A) and Western blot (B). C and D. Inhibition of PDGF-BB stimulated (for 24 h) rat SMC proliferation (BrdU assay)(C) and migration (scratch assay)(D), by BRD4 siRNA knockdown. Quantification: mean ± SEM; n = 3; *P < 0.05 versus scrambled siRNA control.

It has been well documented that proliferation as well as migration of vascular SMCs are major contributors to IH (Alexander and Owens, 2012, Jukema et al., 2012). In response to vascular injury, quiescent SMCs are activated by cytokines or growth factors, migrate into the subintimal space and proliferate, forming highly cellular neointima (Jukema et al., 2012). PDGF signaling is known to be the most potent stimulant for vascular SMC proliferation and migration (Heldin and Westermark, 1999). In our experiments, PDGF-BB stimulation increased BRD4 protein levels (Figure S2). We therefore determined the effect of JQ1(+) on the phenotypic transition of primary rat aortic SMCs stimulated by PDGF-BB. We found that the enantiomer JQ1(−), which is identical to JQ1(+) except being a stereoisomer, had no effect on PDGF-BB-stimulated rat aortic SMC proliferation throughout a range of concentrations from 5 nM to 10 μM. In contrast, JQ1(+) produced a pronounced inhibitory effect in a dose-dependent manner (Fig. 3A). An inhibitory effect of JQ1(+) on SMC proliferation was reproducible using human primary aortic SMCs (Fig. 3B). Importantly, we confirmed that JQ1(+)-treated rat carotid arteries at post-injury day 14 had fewer proliferating cells (PCNA-staining) compared to vehicle control (Fig. 3C). We also found that treatment with 1 μM JQ1(+) inhibited SMC migration (~ 60%) compared to vehicle control whereas JQ1(−) had no effect (Figs. 4 and S3). In support of an important role of BRD4, knocking down BRD4 using an siRNA (Fig. 5, A and B) markedly inhibited rat SMC proliferation (Fig. 5C) as well as migration (Figs. 5D and S4). A specific BRD4 knockdown using BRD4 siRNA was confirmed by the lack of changes in BRD2 and BRD3 mRNA levels (Fig. 5A). These results indicate that blocking BET bromo-domains inhibits SMC proliferation and migration, which at least partly accounts for the abrogation of IH after JQ1(+) treatment (Fig. 2).

### 3.4 BET Inhibition Down-regulates PDGFRα in Cultured SMCs as well as in Balloon-injured Rat Carotid Arteries

**Fig. 6:**
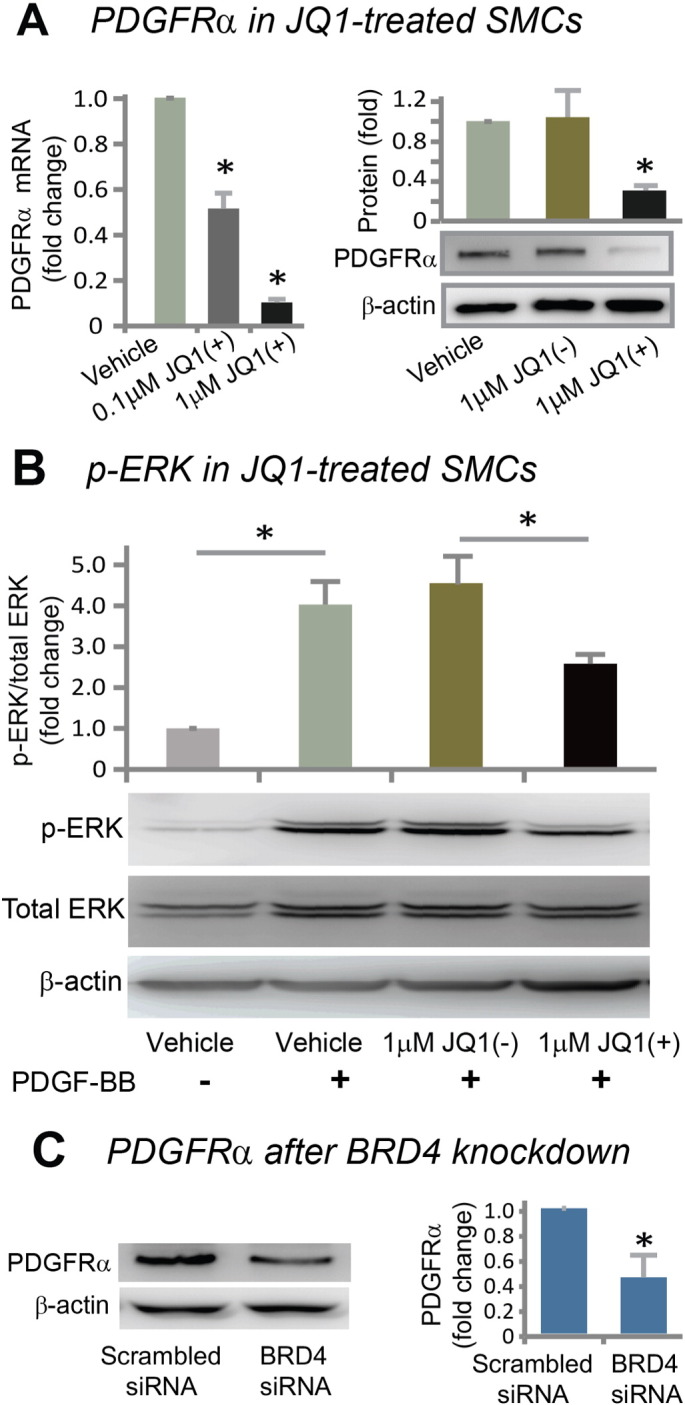
Down-regulation of PDGFRα in vitro by treatment with JQ1(+) or siRNA knockdown of BRD4. A. Determination of PDGFRα mRNA (RT-PCR) and protein (Western blot). mRNA was determined at 6 h and protein was determined at 24 h after treatment with JQ1(+). Quantification: mean ± SEM; n = 3; *P < 0.05 versus vehicle control. B. Inhibition of ERK phosphorylation in SMCs treated with JQ1(+). In order to confirm the effect of JQ1(+) on PDGFR expression, phosphor-ERK, which is a well-established downstream effector and hence a surrogate of PDGFR activation, was determined. Rat aortic SMCs were pretreated with 1 μM JQ1(+), JQ1(−), or equal volume of DMSO for 48 h in starvation medium, as described in Methods, and then PDGF-BB was added (final 10 ng/ml) for stimulation of ERK phosphorylation. Cells were harvested after 15 min and used for Western blotting determinations. Quantification: mean ± SEM; n = 3; *P < 0.05. C. PDGFRα down-regulation by BRD4 siRNA. Quantification: mean ± SEM; n = 3; *P < 0.05 versus scrambled siRNA control.

**Fig. 7:**
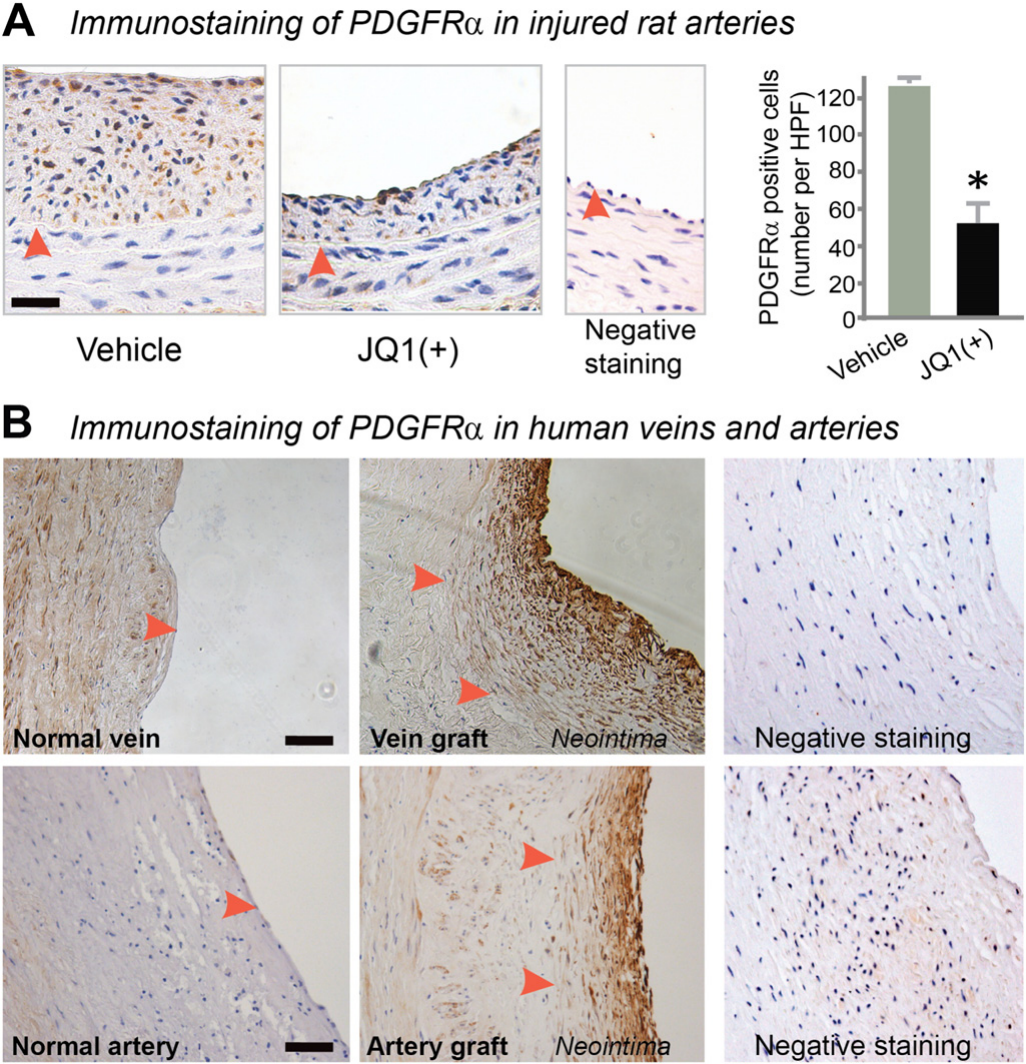
Down-regulation of PDGFRα in vivo by treatment with JQ1(+). A. Immunohistochemistry showing reduced PDGFRα in the media and neointima in JQ1(+)-treated (500 μg per rat) carotid arteries compared to vehicle control 14 days after balloon injury. Arrowhead: IEL. “Negative staining” refers to background control without using a primary antibody. Quantification: mean ± SEM; n = 4 animals; *P < 0.05. HPF: high power field. Scale bar: 50 μm. B. Immunohistochemistry showing increase of PDGFRα in the neointima of human saphenous vein and internal mammary artery grafts compared to normal vessels. Scale bar: 50 μm.

Inasmuch as inhibiting BET activity abrogates PDGF-BB-stimulated SMC proliferation and migration, we focused on PDGF receptors (PDGFRs) as a potential molecular mechanism responsible for the function of the BET family in the SMC phenotypic transition. While 100 nM JQ1(+) reduced PDGFRα mRNA approximately 50% (Fig. 6A), 1 μM JQ1(+), a concentration that produced marked inhibition of both SMC proliferation and migration (see Fig. 3, Fig. 4), almost completely eliminated PDGFRα mRNA and also greatly reduced PDGFRα protein (by 70%). PDGFRβ mRNA but not protein was reduced under the same conditions (Figure S5). Further supporting a BET control over PDGFR expression, 1 μM JQ1(+) significantly suppressed PDGF-BB-stimulated ERK phosphorylation (Fig. 6B) — a well-documented event downstream of PDGFR activation. The functional specificity of JQ1(+) was manifested by the lack of an effect of the JQ1(−) enantiomer on PDGFRs. As well, an important role of BRD4 for the control of PDGFRα expression was supported by up to 60% decrease of PDGFRα protein following siRNA knockdown of BRD4 (Fig. 6C). Furthermore, immunohistochemistry showed less PDGFRα staining in JQ1(+)-treated rat arteries compared to vehicle control at 14 days post injury (Fig. 7A). Interestingly, we found a pattern of post-injury up-regulation of PDGFRα similar to that of BRD4 — increased at day 3 in the media, peaked at day 7 in the neointima, and still maintained at high levels at day 14 in the neointima (Figure S6). In addition, we also observed conspicuously more PDGFRα staining in the neointima of human disease vein and artery grafts compared to normal vessels without hyperplastic neointima (Fig. 7B). These results support a prominent BET control over PDGFRα expression in vitro and in vivo.

### 3.5 BET Inhibition Rescues Vascular Endothelial Cells from Pro-inflammatory Cytokine-induced Dysfunction

**Fig. 8:**
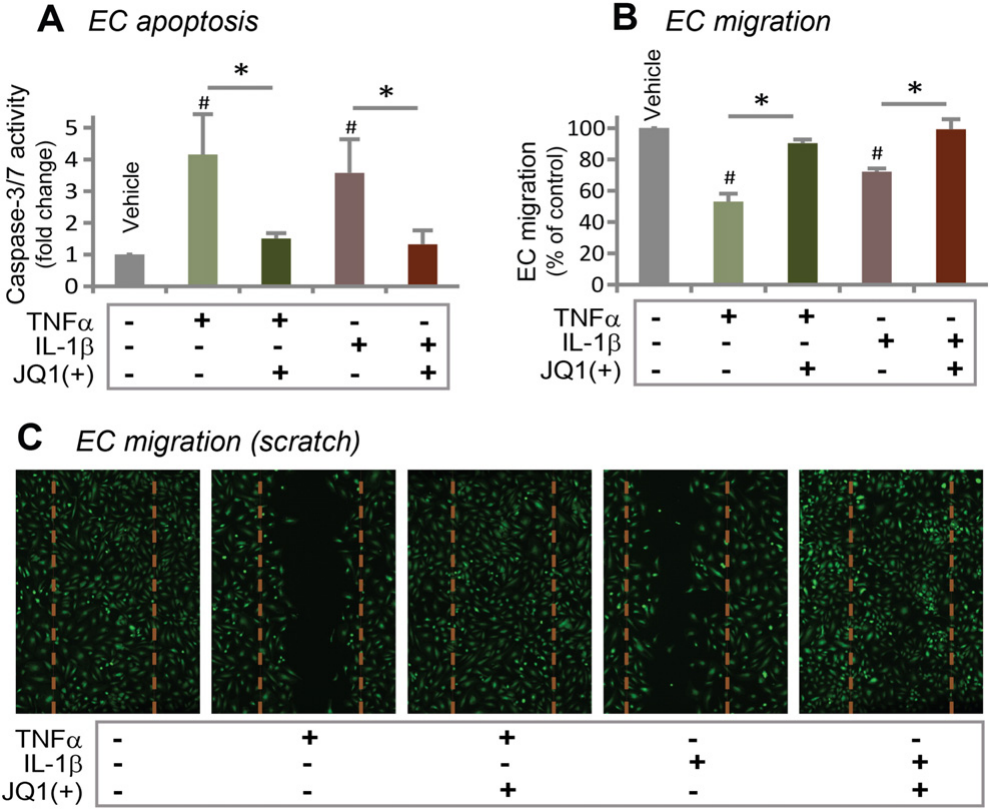
Prevention of inflammatory cytokine-induced vascular endothelial cell dysfunction by JQ1(+). A. TNFα or IL-1β-induced (20 ng/ml, 4 h) human aortic EC apoptosis, measured as Caspase-3/7 activity, was prevented by pre-treatment with 100 nM JQ1(+) for 24 h. B. TNFα or IL-1β-induced (20 ng/ml, 4 h) inhibition of EC migration was prevented by pre-treatment with 100 nM JQ1(+) for 24 h. Quantification: mean ± SEM; n = 3; ^#^P < 0.05 versus vehicle control; *P < 0.05 between the conditions in the absence and presence of JQ1(+). C. Images of human aortic ECs 24 h after scratch. Dashed line marks the cell-free gap at 0 h after scratch. Quantified data are presented in B.

A major drawback of the drugs clinically used in drug-eluting stents (e.g. rapamycin and paclitaxel) for prevention of restenosis is their damaging effects on endothelial cells (ECs) that lead to late-stent thrombosis and sudden death (Iakovou et al., 2005, Inoue et al., 2011). On the other hand, reports of anti-restenotic agents that can preserve EC functions are scarce (Goel et al., 2014). We thus tested the impact of JQ1(+) treatment on human primary aortic ECs. It has been well established that either of the two pro-inflammatory cytokines, TNFα and IL-1β, induces EC apoptosis and inhibits EC migration (Simon, 2012). In our experiment, whereas each of TNFα and IL-1β stimulated Caspase-dependent EC apoptosis by 3–4 fold compared to vehicle control, incubation of the cells with 100 nM JQ1(+) completely prevented this cytokine-induced apoptosis (Fig. 8A). In a separate experiment, whereas either TNFα or IL-1β inhibited EC migration, as determined by scratch assay, incubation with 100 nM JQ(+) restored EC migration (Figs. 8B and S7). These results indicate that BET inhibition protects ECs from functional damage inflicted by pro-inflammatory cytokines.

## 4 Discussion

While the critical role of epigenetic regulators in disease development is becoming widely recognized, epigenetic regulations in vascular diseases, in particular restenosis, are less well understood (Alexander and Owens, 2012). The BET family is a class of epigenetic regulators that “read” histone acetylation and “translate” it into transcriptional activation (Shi and Vakoc, 2014). The dictating role of the BET family in cell identity (Di Micco et al., 2014) as well as cell state transition (Brown et al., 2014) is beginning to be unveiled thanks to the recent discovery of small molecule inhibitors that specifically block the bromodomain/acetyl-lysine interaction (Filippakopoulos and Knapp, 2014). These advances have opened fresh opportunities for therapeutic targeting of BET epigenetic “readers”. However, the role of the BET family in the development of IH — the primary cause of many vasculopathies — is not known (Wang et al., 2015). Here we find that inhibiting the BET family with JQ1(+) or knocking down BRD4 alone abrogates the neointima-promoting SMC transition to a migratory/proliferative cell state while protecting vascular ECs from pro-inflammatory cytokine-induced dysfunction. We have further identified that either BET inhibition or BRD4 knockdown results in down-regulation of PDGFRα, a prominent activator of SMC proliferation and migration. Importantly, using the rat balloon angioplasty model, we find that blocking BET bromodomains with JQ1(+) diminished IH. Thus, our results highlight a potential of targeting the BET family for prevention of IH-associated vascular diseases such as restenosis.

Aside from the pathogenic SMC transition to a migratory/proliferative cell state, dysfunction of ECs also critically contributes to IH (Simon, 2012, Inoue et al., 2011). On the one hand, normal endothelium serving as a protective inner lining shields medial SMCs from various blood-borne stimuli, e.g. growth factors and cytokines, which otherwise stimulate SMC phenotypic transition leading to IH. On the other hand, when activated by inflammatory stimuli, ECs become dysfunctional. They undergo apoptosis and recruit leukocytes, leading to release of inflammatory cytokines that exacerbate SMC pathology and IH. Therefore, the ideal anti-restenotic agent would impair SMC proliferation/migration without affecting EC normal physiology, allowing re-establishment of the endothelium damaged by surgical procedures performed to reopen an atherosclerotic artery (Goel et al., 2014). Unfortunately, the clinically applied anti-restenotic drugs (rapamycin or paclitaxel coated on stents) impair re-endothelialization (Inoue et al., 2011, Wessely et al., 2006), leading to stent thrombosis which is associated with ~ 50% mortality (Iakovou et al., 2005). Agents that inhibit SMC proliferation/migration but preserve EC function have only been scarcely reported, and none has reached clinical trials for prevention of restenosis (Goel et al., 2014). Remarkably, we found that instead of harming ECs, JQ1(+) protected human aortic ECs from TNFα or IL-1β-induced dysfunction (apoptosis and impaired migration). This result is consistent with the finding of Brown et al., i.e., JQ1(+) suppresses TNFα-induced leukoctye recruitment by human umbilical vein ECs(Brown et al., 2014). In addition, our data indicate that treatment with JQ1(+) reduces IH without causing shrinkage of the vessel size (termed constrictive remodeling) which, aside from IH, is another important contributor to restenosis (Guo et al., 2014, Goel et al., 2012). Therefore, targeting BET epigenetic readers appears to be an attractive strategy for prevention of restenosis. Accordantly, in preclinical studies, BET inhibitors have shown extremely low-toxicity (Zuber et al., 2011, Asangani et al., 2014) but high efficacy in treating leukemia (Zuber et al., 2011), multiple myeloma (Delmore et al., 2011) and heart failure (Anand et al., 2013). Some of the inhibitors have entered clinical trials (Wang and Filippakopoulos, 2015).

The BET inhibitors thus far developed are not selective within the BET family. JQ1(+) is highly specific to the BET family but binds to the bromodomains of all family members with similar affinities (Baud et al., 2014). Although we cannot rule out possible contributions of BRD2 and BRD3, our data point to a prominent role of BRD4 in governing SMC proliferation and migration and PDGFRα expression. Evidence includes the following: 1) While specific knockdown of BRD4 using an siRNA reduced SMC proliferation, migration, as well as PDGFRα protein to an extent similar to that of BRD4 reduction (~ 60%), knockdown of BRD2 or BRD3 did not affect SMC proliferation (Figure S8). 2) BRD4 dramatically increased in the rat carotid arterial wall following injury, as well as in the neointima lesion of human vein and artery grafts. 3) In response to PDGF-BB stimulation, BRD4 increased within 30 min, likely due to post-translational regulation, and showed another surge at 6 h, which may reflect de novo protein synthesis. Either BRD2 or BRD3 did not show an mRNA level change over a time course of 24 h (Figure S9). 4) Compared to other BET proteins, BRD4 has a longer sequence containing a unique C-terminal domain that plays a critical role in transcriptional activation by recruiting the positive transcription elongation factor (p-TEFb) (Shi and Vakoc, 2014). 5) As reported by Brown et al., knockdown of BRD4 with an siRNA abolished TNFα-induced EC transition to an inflammatory cell state (Brown et al., 2014). 6) siRNA knockdown or gene silencing identified BRD4 as a potential target to treat a variety of diseases, such as leukemia (Zuber et al., 2011), heart failure (Anand et al., 2013), ovarian carcinoma (Baratta et al., 2015), prostate cancer (Asangani et al., 2014), and pulmonary disease (Khan et al., 2014). Nevertheless, future studies to dissect the functions of BET proteins would be important not only for mechanistic delineation but also optimization of BET-targeted therapeutic paradigms. To this end, the CRISPR (clustered regularly interspaced short palindromic repeats)/Cas9 genome editing technology would be advantageous in completely depleting each of the BET family members (Abrahimi et al., 2015). An exciting latest development is small molecules showing a unique function of tethering BRD4 to an E3 ubiquitin ligase for intracellular destruction, with selectivity over BRD2 and BRD3 (Zengerle et al., 2015, Lu et al., 2015). These reports represent vigorous ongoing efforts to generate selective BET inhibitors that can discriminate different BET members, which would facilitate breakthroughs in both mechanistic and translational studies.

PDGF signaling is the most potent stimulant of vascular SMC state transition to a migratory/proliferative phenotype (Heldin and Westermark, 1999). The BET family (or BRD4) dictates cell state transition, as discovered most recently in ECs and macrophages (Brown et al., 2014). Our results from vascular SMCs indicate that PDGF receptor expression is controlled by BET epigenetic reader activity. First, treatment with JQ1(+) eliminated PDGFRα mRNA and protein. Second, BRD4 knockdown proportionally reduced PDGFRα protein. Furthermore, the time course of post-injury PDGFRα up-regulation in the rat carotid artery showed a pattern similar to that of BRD4. PDGFRα and β are the only receptors for PDGF-BB (Heldin and Westermark, 1999). While PDGFRβ has been intensively studied and shown to play a prominent role in SMC pathology and IH, the specific function of PDGFRα in this context is largely unknown (Li et al., 2011). Our data show a dramatic increase of PDGFRα in the neointima of rat artery as well as human disease vein and artery grafts compared to normal vessels, implicating a role of PDGFRα in the development of IH. Consistently, it was recently reported that PDGF-AA, which binds only to PDGFRα but not PDGFRβ (Heldin and Westermark, 1999), activated PDGFRα and potently stimulated human arterial SMC proliferation (Li et al., 2011). In addition, a neutralizing antibody for PDGFRα reduced baboon vascular SMC proliferation in a concentration-dependent manner (Davies et al., 2000). However, anti-PDGFRβ but not anti-PDGFRα decreased neointima either in injured arteries (Giese et al., 1999) or polytetrafluoroethylene grafts in nonhuman primates (Davies et al., 2000). The authors cautioned that the data on anti-PDGFRα was complicated by rapid clearance of the antibody from circulation (Davies et al., 2000). Thus, elucidation of the role of PDGFRα in neointima formation awaits more sophisticated studies utilizing SMC-specific PDGFRα knockout animals. Nevertheless, we have found a BET-dominated epigenetic regulation of the PDGF signaling. Moreover, differential effects of BET inhibition on the expression of PDGFRα and PDGFRβ support a specific rather than promiscuous BET control over gene expression. Given a critical role of PDGFRα in caner growth and metastasis (Jechlinger et al., 2006), our finding of BRD4 epigenetic control of PDGFRα may lend important insights for cancer research as well.

Taken together, this study demonstrates that blocking BET epigenetic reader activity with JQ1(+) disrupts a cell state transition of SMCs to multiple IH-promoting phenotypes, including proliferation, migration, and inflammation (production of monocyte chemoattractant protein-1, Guo et al., unpublished data), yet protects ECs against cytokine-induced dysfunction. These favorable outcomes may be plausibly rationalized based on the latest breakthroughs in research on epigenetic mechanisms. Super-enhancers, or genomic DNA units containing clustered enhancers (Pott and Lieb, 2015), have been discovered to be key determinants of cell fate or cell state transition (Whyte et al., 2013). They amass a large proportion of epigenetic factors including BRD4, master transcription factors, mediators (e.g. Med1), enhancer chromatin marks (e.g. H3K27Ac), as well as RNA polymerase II (Hnisz et al., 2013). Super-enhancers are found to be associated with BRD4 at overactive genes in several pathologies, e.g. cancer progression (Whyte et al., 2013, Loven et al., 2013), EC activation (Brown et al., 2014), and cardiomyocyte hypertrophy (Anand et al., 2013). Upon pathogenic stimulation, super-enhancers re-assemble at a subset of overactive genes promoting their transcription which drives cell state transition (Brown et al., 2014). Importantly, transcription elongation cannot be activated until BRD4 “reads” (or binds) histone acetyl-lysine and then recruits p-TEFb which in turn phosphorylates and activates RNA polymerase II (Shi and Vakoc, 2014). Thus BRD4 provides a sensitive intervention target, blockade of which disrupts super-enhancers and associated pathogenic cell state transition. It is therefore plausible to propose that in our study JQ1(+) treatment might have preferentially suppressed the expression of overactive genes involved in SMC transition to a migratory/proliferative state and EC transition to a inflammatory state, by breaking the assembly of BRD4/acetyl-histone/super-enhancers. Our ongoing studies of ChIP sequencing and RNA sequencing using SMCs are expected to reveal more target genes beyond PDGF receptors that are critically regulated by BRD4 or the BET family. It is noteworthy that c-Myc, a master transcription factor recently found to be the major target of BRD4 in cancer cells (Delmore et al., 2011), was not altered in rat SMCs following JQ1(+) treatment (Guo et al., unpublished data), highlighting a cell type specificity of BET regulations.

## 5 Study Limitations

Although we observed pronounced up-regulation of BRD4 in the neointima of human vein and artery grafts versus normal vessels, the effect of JQ1(+) in healthy rat carotid arteries may be different from that in diseased human vessels, which may limit the ability to extrapolate the current findings to outcomes in humans. In addition, an in vivo test of the effect of JQ1(+) on the recovery of damaged endothelium would lead to more in-depth understanding of the therapeutic potential of the BET-targeting strategy. It should also be noted that the siRNA knockdown of BRD2 was inefficient and may be insufficient to rule out functional effects of this protein. Thus further investigations are warranted to delineate the specific function of BRD2 in SMC pathophysiology. Comparative studies to evaluate the contributions of all three BET members in the development of neointima would assist the development of optimal BET inhibitors for therapeutic applications.

## 6 Conclusion

This study demonstrates that IH can be abrogated via inhibition of BET epigenetic reader activity. We have further identified that the BET family controls the expression of PDGF receptors — the most potent stimulant of the IH-promoting SMC transition to a migratory/proliferative cell state. In light of the human relevance of these findings and the recent progress that some BET inhibitors have entered clinical trials, targeting BET bromodomains appears to be a promising strategy for treating vascular diseases typified by IH.

## Conflicts of Interest

The authors declare no conflicts of interest.
