# Supplementary material for: BET Bromodomain Blockade Mitigates Intimal Hyperplasia in Rat Carotid Arteries
Source: eBioMedicine. 2015 Sep 28;2(11):1650–61. doi: 10.1016/j.ebiom.2015.09.045 (PMC4740308; doi:10.1016/j.ebiom.2015.09.045)

Supplemental figures

Figure legends

Figure S1. BRD4 immunostaining and Van Gieson staining of human veins and arteries

In order to confirm the anatomical neointimal localization of the intense immunostaining of BRD4 (refer to Figure 1) in human saphenous vein grafts and internal mammary artery grafts (in contrast to normal human vein and artery specimens), Van Gieson staining was performed on the sections adjacent to the ones used for BRD4 immunostaining. Shown are Van Gieson-stained distinct medial and neointimal layers. Arrowhead marks the internal elastic lamina (IEL). Scale bar: 50 μm.

Figure S2. PDGF-BB stimulated BRD4 up-regulation in rat aortic smooth muscle cells

A. Rat aortic SMCs were treated with either solvent control or PDGF-BB (final 10 ng/ml), and cells were collected at the indicated time points for BRD4 Western blotting assay.

B. Quantification: mean $\pm$ SEM; n = 3 independent experiments; *P<0.05 compared to solvent control.

Figure S3. Zero hour images of scratch cell migration assay

Bright-field images were taken immediately after a cell-free gap was made by scratching the confluent SMC layer on the bottom of the well, as described in Methods. The same cell culture plates were imaged after 24h incubation (see Figure 4A).

Figure S4. Zero hour images of scratch cell migration assay

Bright-field images were taken immediately after a cell-free gap was made by scratching the confluent SMC layer on the bottom of the well, as described in Methods. The same cell culture plates were imaged after 24h incubation (see Figure 5D).

Figure S5. PDGFRβ expression after treatment with JQ1(+) in vitro

Levels of PDGFRβ mRNA and protein were determined by RT-PCR and Western blotting, respectively, using rat aortic SMCs treated with JQ1(+), JQ1(-), or equal volume of DMSO. RT-PCR was determined for cells after 6h treatment; Western blotting was performed with cells treated for 24h. Quantification is presented as mean ± SEM; n=3 independent experiments; *P<0.05 compared to vehicle control.

Figure S6. Time course of post-injury PDGFRα up-regulation in the rat carotid artery

Immunohistochemistry on rat carotid artery sections showing increase of PDGFRα at days 3, 7, and 14 after balloon angioplasty. "Negative staining" refers to background control without using a primary antibody. Arrowhead: internal elastic lamina (IEL). Neointima is defined between lumen and IEL. Scale bar: 50 μm.

Figure S7. Zero hour images of human endothelial cell migration assay

Bright-field images were taken immediately after a cell-free gap was made by scratching the confluent human aortic EC layer on the bottom of the well, as described in Methods. The same cell culture plates were imaged after 24h incubation (see Figure 8).

Figure S8. Effect of siRNA knockdown of BRD2 and BRD3 on rat smooth muscle cell proliferation

BRD2 (A) or BRD3 (B) was knocked down using siRNAs as described in Methods. SMC proliferation (BrdU assay) was performed 24h after PDGF-BB stimulation. Specificity of knockdown is indicated by a lack of changes in mRNA levels of other BET members. Quantification: mean ± SEM; n=3; *P<0.05 *versus* scrambled siRNA control.

Figure S9. Time courses of BRD2 and BRD3 expression after PDGF-BB stimulation

BRD2 (A) and BRD3 (B) mRNA levels were measured by RT-PCR at indicated time points after PDGF-BB treatment of rat aortic SMCs. Control: solvent; PDGF-BB:10 ng/ml. Quantification: mean ± SEM; n = 3 independent experiments.

Figure S1

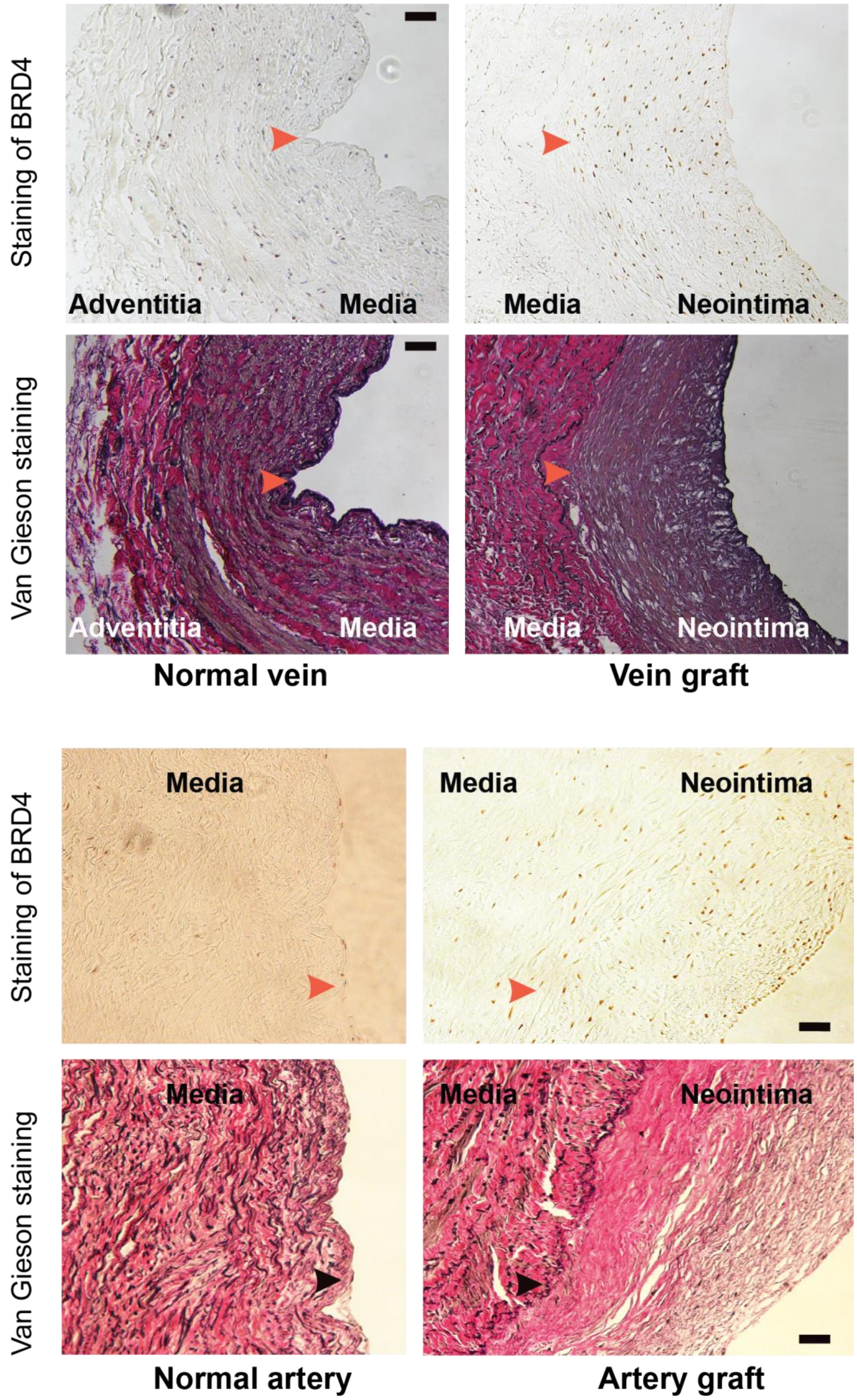

Staining of BRD4
Van Gieson staining
Adventitia
Media
Neointima
Normal vein
Vein graft
Staining of BRD4
Van Gieson staining
Media
Neointima
Normal artery
Artery graft

Figure S2

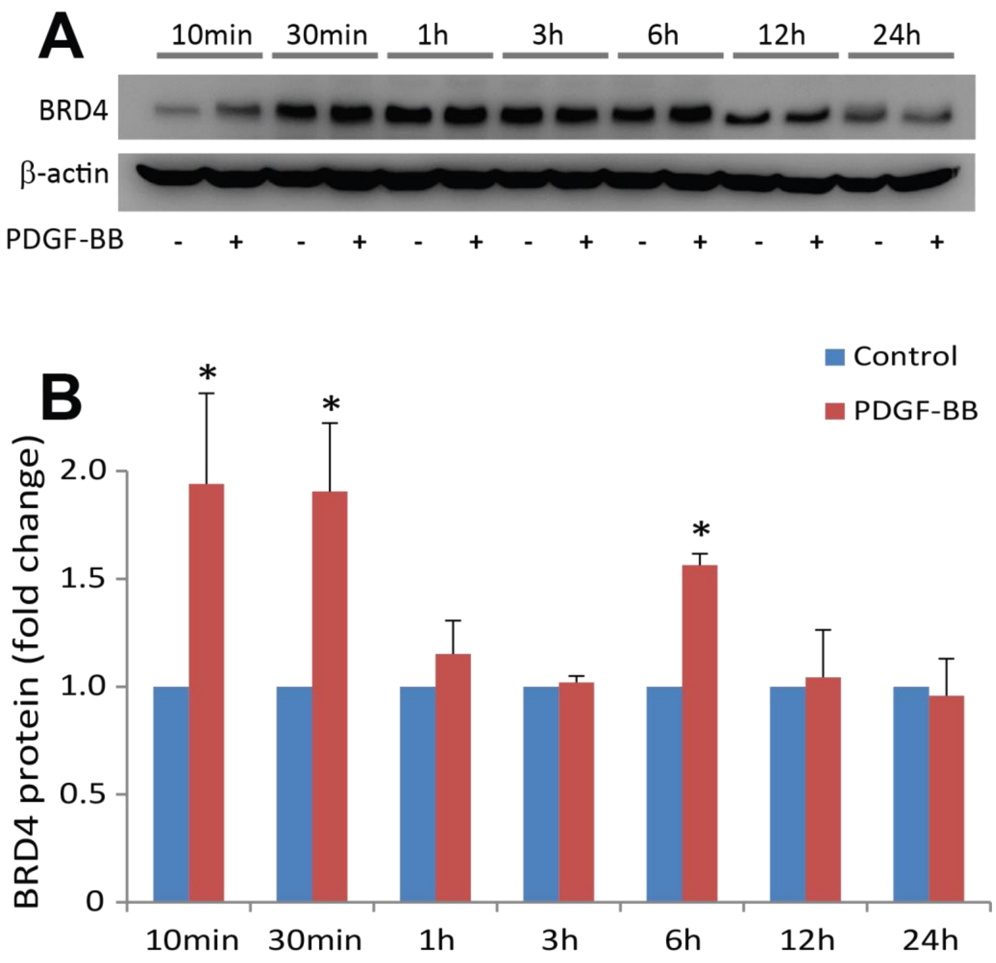
A
10min
30min
1h
3h
6h
12h
24h
BRD4
β-actin
PDGF-BB
- + - + - + - + - + - + - +
B
Control
PDGF-BB
BRD4 protein (fold change)
2.0
1.5
1.0
0.5
0
*
*
*
10min
30min
1h
3h
6h
12h
24h

Figure S3

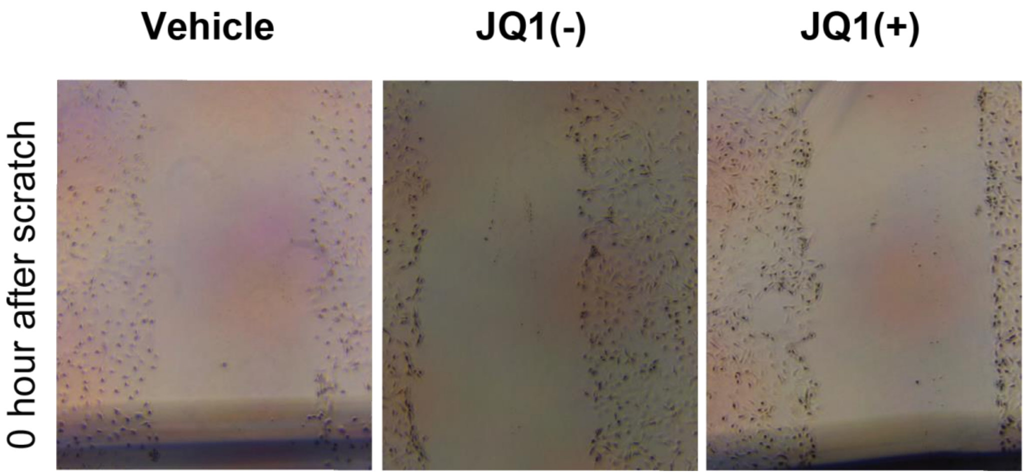

Figure S4

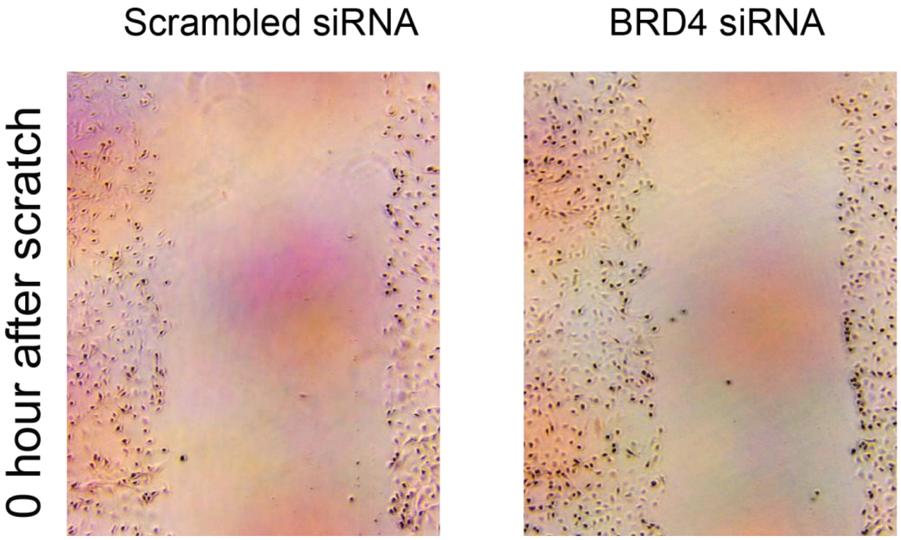

Figure S5

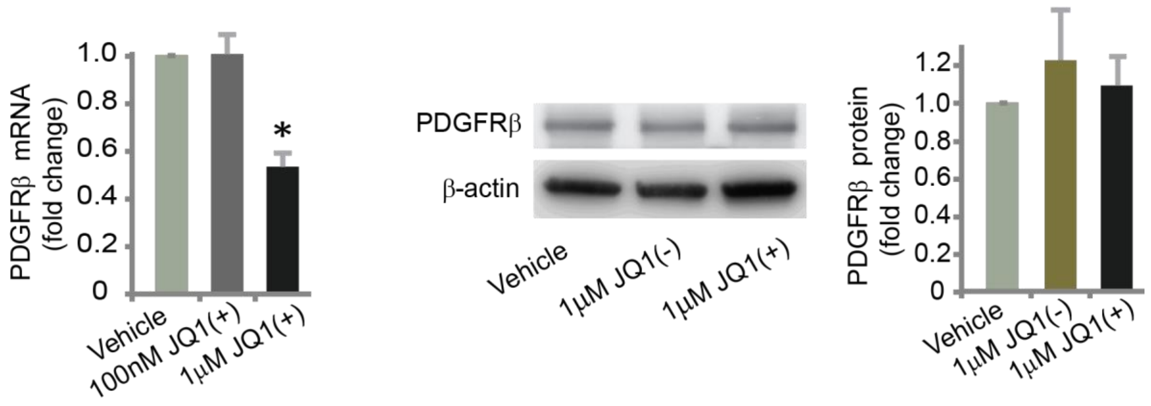
PDGFRβ mRNA
(fold change)
1.0
0.8
0.6
0.4
0.2
0
*
Vehicle
100nM JQ1(+)
1μM JQ1(+)
PDGFRβ
β-actin
Vehicle
1μM JQ1(-)
1μM JQ1(+)
PDGFRβ protein
(fold change)
1.2
1.0
0.8
0.6
0.4
0.2
0
Vehicle
1μM JQ1(-)
1μM JQ1(+)

Figure S6

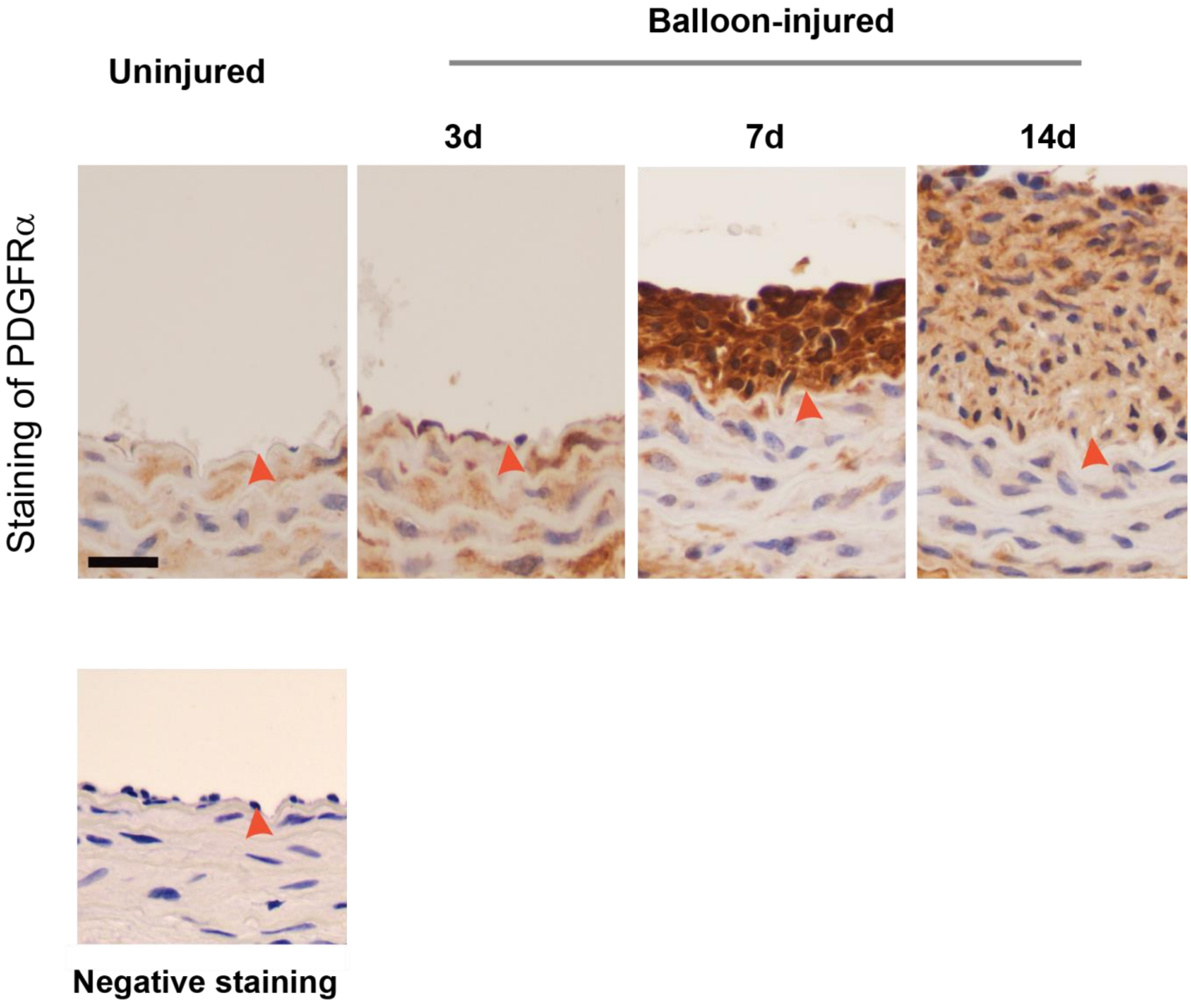
Balloon-injured
Uninjured
3d
7d
14d
Staining of PDGFRα
Negative staining

Figure S7

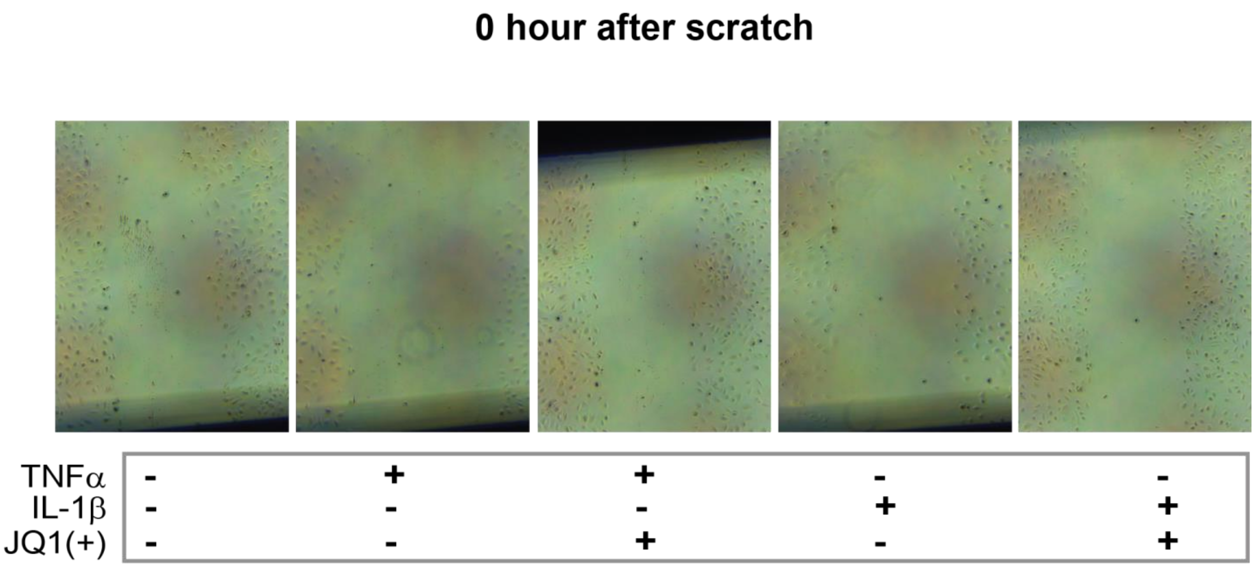

Figure S8

A BRD2 knockdown

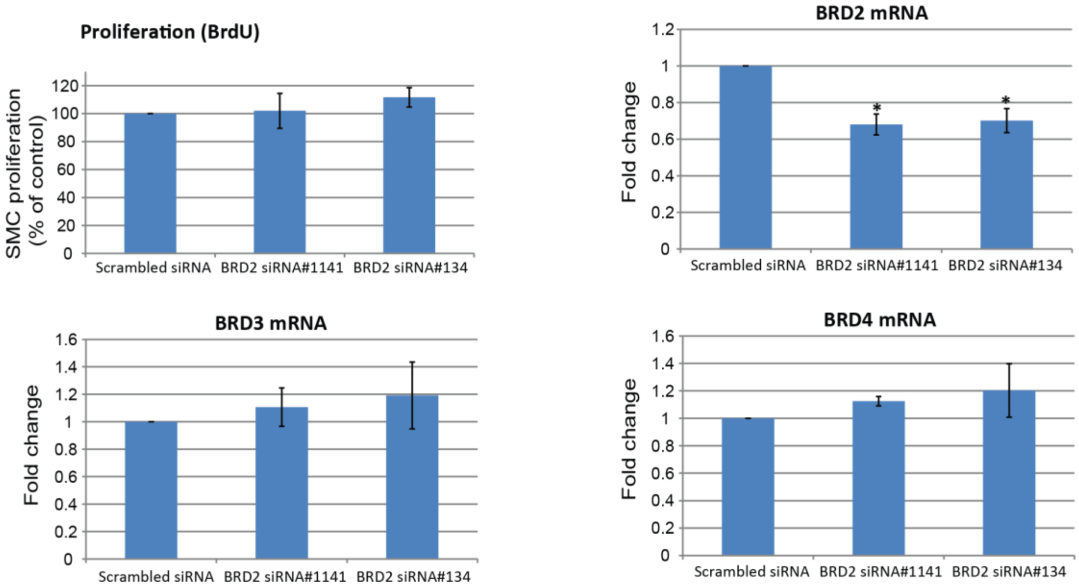

B BRD3 knockdown

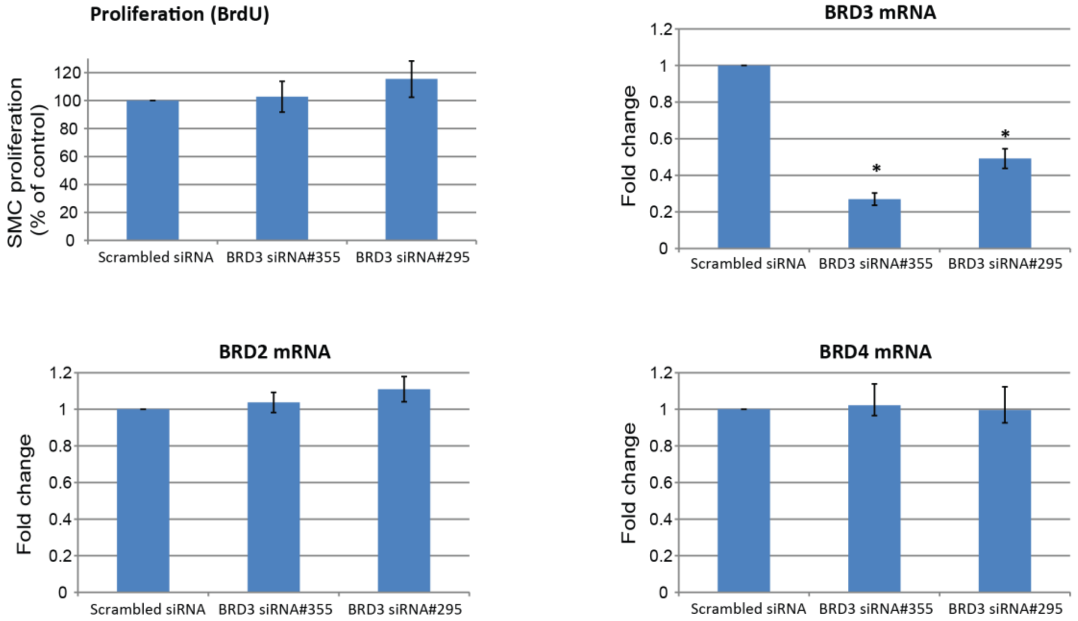

Figure S9

A **BRD2**

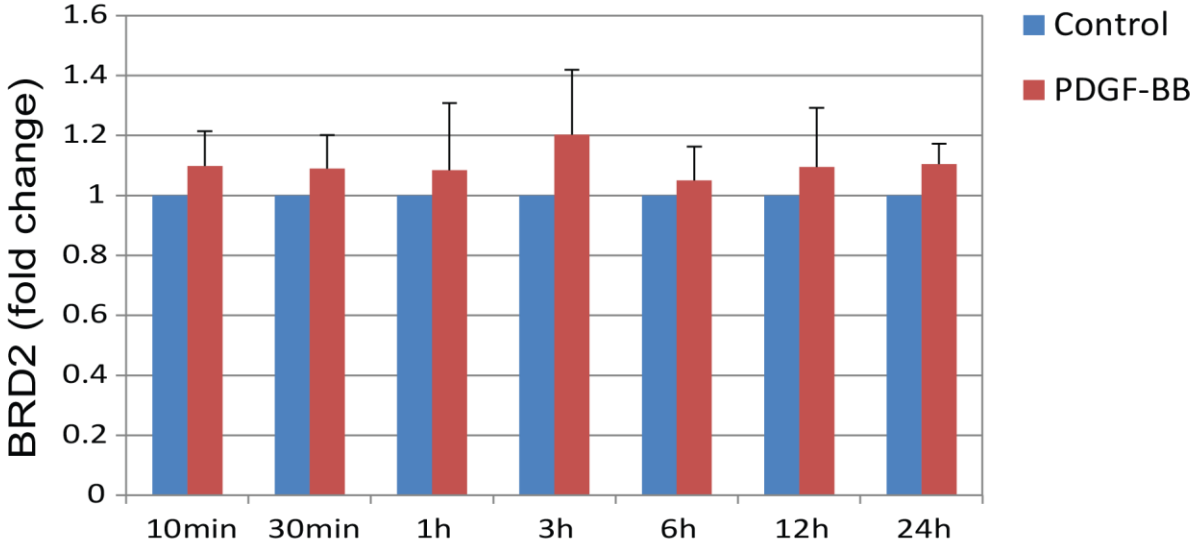

B **BRD3**

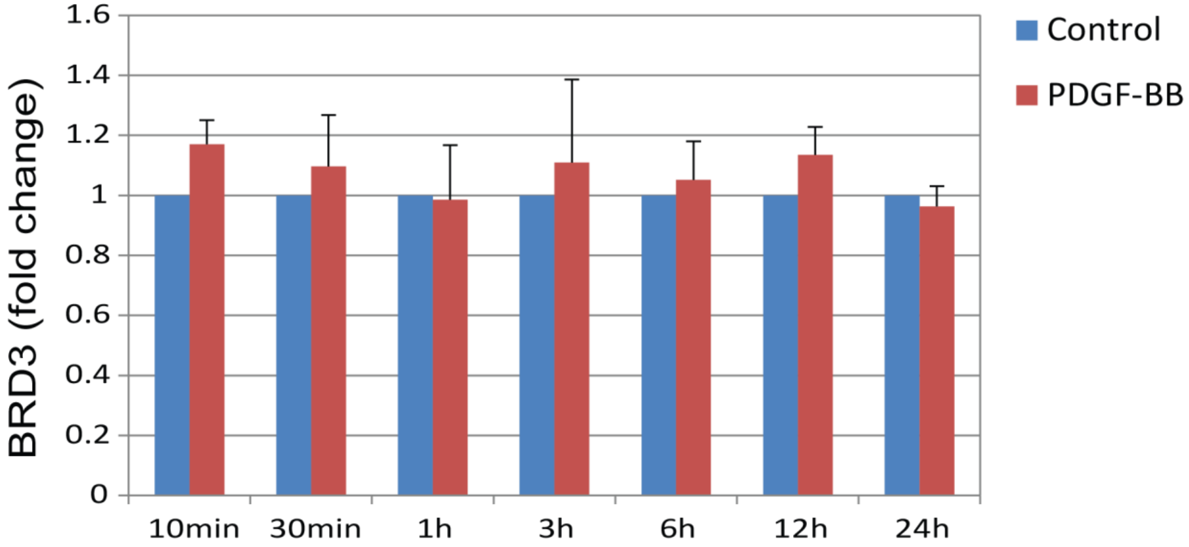

Supplement: Supplementary file 1 — Supplementary data component 1 [file mmc1.pdf]
